# 2-(2-Benzyl­phen­yl)propan-2-ol

**DOI:** 10.1107/S1600536811047143

**Published:** 2011-11-12

**Authors:** Richard Betz, Thomas Gerber, Eric Hosten, B. P. Siddaraju, Hemmige S. Yathirajan, A. R. Ramesha

**Affiliations:** a Nelson Mandela Metropolitan University, Summerstrand Campus, Department of Chemistry, University Way, Summerstrand, PO Box 77000, Port Elizabeth 6031, South Africa; b University of Mysore, Department of Studies in Chemistry, Manasagangotri, Mysore 570 006, India; c R. L. Fine Chem., Bangalore 560 064, India

## Abstract

There are two mol­ecules in the asymmetric unit of the title compound, C_16_H_18_O, a tertiary alcohol featuring a 2-benzyl­phenyl substituent. Co-operative O—H⋯O hydrogen bonds connect the mol­ecules into tetra­mers.

## Related literature

For general background to the use of benzhydrols in pharmaceutical synthesis, see: Ohkuma *et al.* (2000 ▶). For related structures, see: Ferguson *et al.* (1995 ▶); Fun *et al.* (2010 ▶); Siddaraju *et al.* (2010 ▶, 2011 ▶); Zeng & Liu (2010 ▶); Gu *et al.* (2009 ▶). For graph-set analysis of hydrogen bonds, see: Etter *et al.* (1990 ▶); Bernstein *et al.* (1995 ▶).
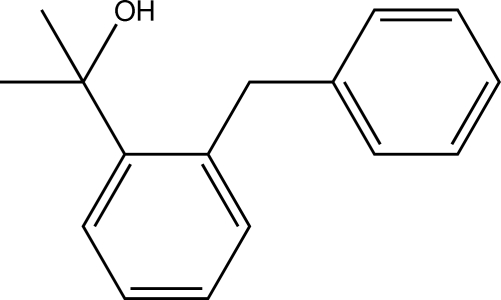


## Experimental

### 

#### Crystal data

C_16_H_18_O
*M* *_r_* = 226.30
Monoclinic, 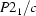

*a* = 12.2252 (3) Å
*b* = 17.2508 (4) Å
*c* = 16.7784 (3) Å
β = 132.549 (1)°
*V* = 2606.79 (10) Å^3^
*Z* = 8
Mo *K*α radiation
μ = 0.07 mm^−1^
*T* = 200 K
0.59 × 0.51 × 0.34 mm

#### Data collection

Bruker APEXII CCD diffractometer
Absorption correction: multi-scan (*SADABS*; Bruker, 2008 ▶) *T*_min_ = 0.901, *T*_max_ = 1.000
24429 measured reflections
6480 independent reflections
5035 reflections with *I* > 2σ(*I*)
*R*_int_ = 0.015

#### Refinement

*R*[*F* ^2^ > 2σ(*F* ^2^)] = 0.053
*wR*(*F* ^2^) = 0.148
*S* = 1.05
6480 reflections
311 parameters
H-atom parameters constrained
Δρ_max_ = 0.67 e Å^−3^
Δρ_min_ = −0.57 e Å^−3^

### 

Data collection: *APEX2* (Bruker, 2010 ▶); cell refinement: *SAINT* (Bruker, 2010 ▶); data reduction: *SAINT*; program(s) used to solve structure: *SHELXS97* (Sheldrick, 2008 ▶); program(s) used to refine structure: *SHELXL97* (Sheldrick, 2008 ▶); molecular graphics: *ORTEP-3* (Farrugia, 1997 ▶) and *Mercury* (Macrae *et al.*, 2008 ▶); software used to prepare material for publication: *SHELXL97* and *PLATON* (Spek, 2009 ▶).

## Supplementary Material

Crystal structure: contains datablock(s) I, global. DOI: 10.1107/S1600536811047143/hg5130sup1.cif

Supplementary material file. DOI: 10.1107/S1600536811047143/hg5130Isup2.cdx

Structure factors: contains datablock(s) I. DOI: 10.1107/S1600536811047143/hg5130Isup3.hkl

Supplementary material file. DOI: 10.1107/S1600536811047143/hg5130Isup4.cml

Additional supplementary materials:  crystallographic information; 3D view; checkCIF report

## Figures and Tables

**Table 1:** Hydrogen-bond geometry (Å, °)

*D*—H⋯*A*	*D*—H	H⋯*A*	*D*⋯*A*	*D*—H⋯*A*
O1—H1⋯O2^i^	0.84	1.98	2.7997 (14)	166
O2—H2⋯O1	0.84	1.94	2.7486 (14)	161

Symmetry code: (i) 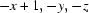.

**Fig. 1.**
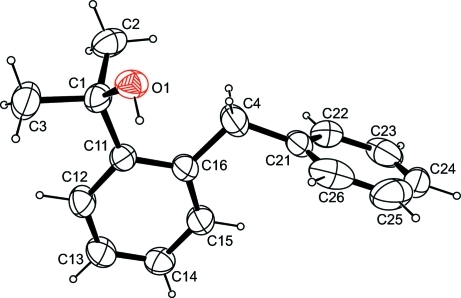
The molecular structure of the title compound, with atom labels and anisotropic displacement ellipsoids (drawn at 50% probability level). For clarity, only one of the molecules present in the asymmetric unit is shown.

**Fig. 2.**
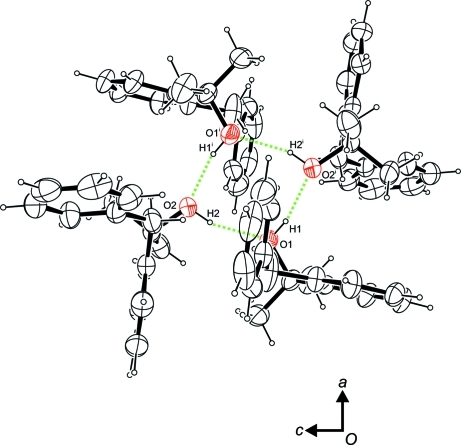
Intermolecular contacts, viewed along [0 1 0]. Symmetry operator: i -x + 1, -y, -z.

**Fig. 3.**
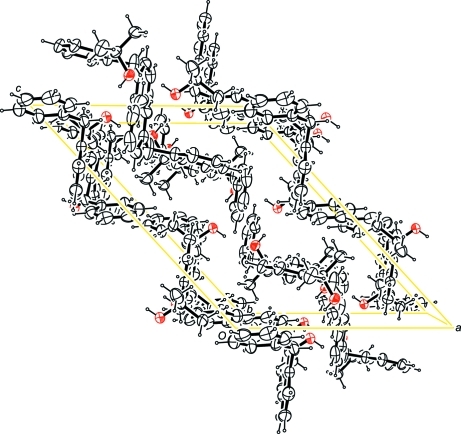
Molecular packing of the title compound, viewed along [0 1 0] (anisotropic displacement ellipsoids drawn at 50% probability level).

## supplementary crystallographic information

### Comment

2-(2-Benzylphenyl)propan-2-ol is used in synthetic organic chemistry for the preparation of many organic compounds including anthrone. Benzhydrols are widely used as intermediates for the synthesis of pharmaceuticals (Ohkuma *et al.*, 2000). The crystal structures of 2-(5-bromo-2-methylphenyl)propan-2-ol (Zeng & Liu, 2010), 10,10-dimethylanthrone (Fun *et al.*, 2010), (2-methylphenyl)(phenyl)methanol (Siddaraju *et al.*, 2010), 9,9-dimethyl-9,10-dihydroanthracene (Siddaraju *et al.*, 2011) and a *N,N*-dimethylamino-substituted analogue of the title compound (Gu *et al.*, 2009) have been reported earlier. In view of the importance of the title compound, its crystal structure was determined.

The asymmetric unit contains two complete molecules. The least-squares planes defined by the carbon atoms of the different phenyl moieties in each molecule enclose angles of 82.58 (10)° and 88.66 (13)°, respectively (Fig. 1).

In the crystal, cooperative hydrogen bonds connect the molecules to discrete tetramers. The plane defined by the atoms of the participating hydroxyl groups is perpendicular to the crystallographic *b* axis. In terms of graph-set analysis (Etter *et al.*, 1990; Bernstein *et al.*, 1995), the descriptor for the hydrogen bonds is *DD* on the unitary level and *R*^4^_4_(8) on the binary level. The shortest intercentroid distance between two aromatic systems was measured at 4.9147 (14) Å and is observed between the two different phenyl rings of one of the molecules present in the asymmetric unit and its symmetry-generated equivalent (Fig. 2).

The packing of the title compound in the crystal is shown in Figure 3.

### Experimental

The title compound was obtained as a gift sample from *R. L*. Fine Chem, Bangalore, India. X-ray quality crystals were obtained by slow evaporation from toluene solution at room temperature.

### Refinement

Carbon-bound H atoms were placed in calculated positions (C—H 0.95 Å for aromatic carbon atoms, C—H 0.99 Å for methylene groups) and were included in the refinement in the riding model approximation, with *U*(H) set to 1.2*U*_eq_(C). The H atoms of the methyl groups were allowed to rotate with a fixed angle around the C—C bond to best fit the experimental electron density (HFIX 137 in the *SHELX* program suite (Sheldrick, 2008), with *U*(H) set to 1.5*U*_eq_(C). Both oxygen-bound H atoms were placed in calculated positions (O—H 0.94 Å) and were included in the refinement in the riding model approximation, with *U*(H) set to 1.5*U*_eq_(O).

### Crystal data

**Table:**

C_16_H_18_O	*F*(000) = 976
*M**_r_* = 226.30	*D*_x_ = 1.153 Mg m^−^^3^
Monoclinic, *P*2_1_/*c*	Melting point = 333–335 K
Hall symbol: -P 2ybc	Mo *K*α radiation, λ = 0.71073 Å
*a* = 12.2252 (3) Å	Cell parameters from 9880 reflections
*b* = 17.2508 (4) Å	θ = 2.6–28.3°
*c* = 16.7784 (3) Å	µ = 0.07 mm^−^^1^
β = 132.549 (1)°	*T* = 200 K
*V* = 2606.79 (10) Å^3^	Block, colourless
*Z* = 8	0.59 × 0.51 × 0.34 mm

### Data collection

**Table:**

Bruker APEXII CCD diffractometer	6480 independent reflections
Radiation source: fine-focus sealed tube	5035 reflections with *I* > 2σ(*I*)
graphite	*R*_int_ = 0.015
φ and ω scans	θ_max_ = 28.3°, θ_min_ = 2.0°
Absorption correction: multi-scan (*SADABS*; Bruker, 2008)	*h* = −16→16
*T*_min_ = 0.901, *T*_max_ = 1.000	*k* = −23→21
24429 measured reflections	*l* = −22→20

### Refinement

**Table:**

Refinement on *F*^2^	Primary atom site location: structure-invariant direct methods
Least-squares matrix: full	Secondary atom site location: difference Fourier map
*R*[*F*^2^ > 2σ(*F*^2^)] = 0.053	Hydrogen site location: inferred from neighbouring sites
*wR*(*F*^2^) = 0.148	H-atom parameters constrained
*S* = 1.05	*w* = 1/[σ^2^(*F*_o_^2^) + (0.0593*P*)^2^ + 0.964*P*] where *P* = (*F*_o_^2^ + 2*F*_c_^2^)/3
6480 reflections	(Δ/σ)_max_ < 0.001
311 parameters	Δρ_max_ = 0.67 e Å^−^^3^
0 restraints	Δρ_min_ = −0.57 e Å^−^^3^

### Fractional atomic coordinates and isotropic or equivalent isotropic displacement parameters (Å^2^)

**Table:**

	*x*	*y*	*z*	*U*_iso_*/*U*_eq_	
O1	0.57510 (11)	−0.02752 (6)	0.13404 (8)	0.0415 (2)	
H1	0.4865	−0.0134	0.0848	0.062*	
C1	0.61107 (17)	−0.03064 (8)	0.23555 (12)	0.0402 (3)	
C2	0.77983 (18)	−0.02453 (11)	0.32472 (13)	0.0528 (4)	
H2A	0.8256	−0.0677	0.3183	0.079*	
H2B	0.8096	−0.0267	0.3955	0.079*	
H2C	0.8126	0.0247	0.3176	0.079*	
C3	0.5637 (2)	−0.11099 (9)	0.24153 (17)	0.0610 (5)	
H3A	0.4554	−0.1156	0.1850	0.092*	
H3B	0.5955	−0.1179	0.3126	0.092*	
H3C	0.6098	−0.1509	0.2308	0.092*	
C4	0.6403 (3)	0.13895 (10)	0.2002 (2)	0.0778 (7)	
H4A	0.7456	0.1355	0.2687	0.093*	
H4B	0.6259	0.1020	0.1487	0.093*	
C11	0.53379 (15)	0.03600 (8)	0.24184 (11)	0.0357 (3)	
C12	0.45154 (19)	0.01992 (9)	0.26930 (13)	0.0477 (4)	
H12	0.4452	−0.0323	0.2840	0.057*	
C13	0.3783 (2)	0.07688 (10)	0.27620 (14)	0.0505 (4)	
H13	0.3235	0.0637	0.2956	0.061*	
C14	0.38553 (18)	0.15206 (9)	0.25495 (13)	0.0458 (3)	
H14	0.3343	0.1916	0.2579	0.055*	
C15	0.46826 (19)	0.16975 (9)	0.22906 (15)	0.0499 (4)	
H15	0.4744	0.2223	0.2155	0.060*	
C16	0.54339 (17)	0.11350 (8)	0.22197 (13)	0.0428 (3)	
C21	0.61156 (17)	0.21996 (8)	0.15524 (13)	0.0428 (3)	
C22	0.70386 (17)	0.27937 (10)	0.22356 (13)	0.0495 (4)	
H22	0.7828	0.2697	0.2987	0.059*	
C23	0.6818 (2)	0.35404 (11)	0.1827 (2)	0.0677 (6)	
H23	0.7459	0.3952	0.2294	0.081*	
C24	0.5661 (3)	0.36713 (14)	0.0742 (2)	0.0802 (8)	
H24	0.5495	0.4177	0.0455	0.096*	
C25	0.4755 (3)	0.30789 (18)	0.00804 (18)	0.0823 (8)	
H25	0.3949	0.3173	−0.0669	0.099*	
C26	0.49900 (19)	0.23568 (14)	0.04793 (15)	0.0637 (5)	
H26	0.4355	0.1948	0.0000	0.076*	
O2	0.70453 (11)	−0.02771 (7)	0.04946 (8)	0.0462 (3)	
H2	0.6841	−0.0321	0.0880	0.069*	
C5	0.85971 (15)	−0.04565 (9)	0.11354 (12)	0.0438 (3)	
C6	0.8862 (2)	−0.04113 (13)	0.03673 (15)	0.0625 (5)	
H6A	0.8188	−0.0769	−0.0239	0.094*	
H6B	0.9892	−0.0555	0.0757	0.094*	
H6C	0.8677	0.0119	0.0091	0.094*	
C7	0.8837 (2)	−0.12903 (10)	0.15236 (17)	0.0607 (5)	
H7A	0.8622	−0.1333	0.1989	0.091*	
H7B	0.9870	−0.1440	0.1933	0.091*	
H7C	0.8172	−0.1634	0.0898	0.091*	
C8	0.81113 (17)	0.13174 (10)	0.07974 (14)	0.0516 (4)	
H8A	0.7657	0.0919	0.0226	0.062*	
H8B	0.7324	0.1520	0.0765	0.062*	
C31	0.95608 (15)	0.01350 (9)	0.20631 (11)	0.0376 (3)	
C32	1.07226 (16)	−0.01306 (10)	0.31153 (12)	0.0449 (3)	
H32	1.0915	−0.0671	0.3237	0.054*	
C33	1.16028 (18)	0.03729 (11)	0.39872 (13)	0.0538 (4)	
H33	1.2398	0.0179	0.4693	0.065*	
C34	1.1315 (2)	0.11554 (12)	0.38212 (14)	0.0579 (4)	
H34	1.1888	0.1504	0.4415	0.069*	
C35	1.01901 (19)	0.14296 (10)	0.27878 (14)	0.0530 (4)	
H35	1.0008	0.1972	0.2679	0.064*	
C36	0.93098 (16)	0.09365 (9)	0.18956 (12)	0.0416 (3)	
C41	0.86805 (17)	0.19740 (9)	0.05614 (12)	0.0436 (3)	
C42	0.7984 (2)	0.26888 (10)	0.02209 (14)	0.0553 (4)	
H42	0.7161	0.2775	0.0159	0.066*	
C43	0.8475 (3)	0.32829 (10)	−0.00309 (15)	0.0645 (5)	
H43	0.7988	0.3771	−0.0261	0.077*	
C44	0.9662 (2)	0.31676 (11)	0.00512 (14)	0.0609 (5)	
H44	0.9992	0.3573	−0.0127	0.073*	
C45	1.0364 (2)	0.24642 (12)	0.03907 (15)	0.0602 (5)	
H45	1.1188	0.2382	0.0452	0.072*	
C46	0.98818 (18)	0.18725 (10)	0.06445 (14)	0.0523 (4)	
H46	1.0381	0.1387	0.0880	0.063*	

### Atomic displacement parameters (Å^2^)

**Table:**

	*U*^11^	*U*^22^	*U*^33^	*U*^12^	*U*^13^	*U*^23^
O1	0.0422 (5)	0.0485 (6)	0.0408 (5)	0.0068 (4)	0.0309 (5)	0.0031 (4)
C1	0.0500 (8)	0.0371 (7)	0.0435 (7)	0.0075 (6)	0.0357 (7)	0.0070 (6)
C2	0.0503 (9)	0.0608 (10)	0.0449 (8)	0.0198 (8)	0.0312 (8)	0.0135 (7)
C3	0.0907 (14)	0.0371 (8)	0.0856 (13)	0.0084 (8)	0.0718 (12)	0.0095 (8)
C4	0.1053 (16)	0.0417 (9)	0.155 (2)	0.0107 (10)	0.1157 (18)	0.0167 (11)
C11	0.0390 (7)	0.0374 (7)	0.0348 (6)	0.0026 (5)	0.0266 (6)	0.0025 (5)
C12	0.0650 (10)	0.0435 (8)	0.0574 (9)	0.0039 (7)	0.0505 (9)	0.0076 (7)
C13	0.0627 (10)	0.0563 (9)	0.0594 (9)	0.0027 (8)	0.0521 (9)	0.0028 (7)
C14	0.0511 (8)	0.0482 (8)	0.0511 (8)	0.0061 (7)	0.0397 (8)	−0.0009 (7)
C15	0.0612 (10)	0.0369 (7)	0.0721 (11)	0.0035 (7)	0.0534 (9)	0.0023 (7)
C16	0.0488 (8)	0.0370 (7)	0.0592 (9)	0.0019 (6)	0.0431 (8)	0.0027 (6)
C21	0.0469 (8)	0.0382 (7)	0.0617 (9)	0.0000 (6)	0.0441 (8)	0.0013 (6)
C22	0.0394 (8)	0.0564 (9)	0.0490 (8)	0.0028 (7)	0.0284 (7)	−0.0002 (7)
C23	0.0699 (12)	0.0452 (9)	0.1210 (18)	−0.0125 (9)	0.0779 (14)	−0.0186 (10)
C24	0.1033 (17)	0.0689 (13)	0.131 (2)	0.0483 (13)	0.1041 (18)	0.0576 (14)
C25	0.0711 (13)	0.130 (2)	0.0627 (12)	0.0475 (15)	0.0519 (12)	0.0401 (14)
C26	0.0414 (8)	0.0981 (15)	0.0513 (9)	−0.0025 (9)	0.0312 (8)	−0.0142 (10)
O2	0.0325 (5)	0.0694 (7)	0.0390 (5)	−0.0031 (5)	0.0250 (4)	−0.0037 (5)
C5	0.0329 (7)	0.0575 (9)	0.0430 (7)	−0.0033 (6)	0.0264 (6)	−0.0094 (6)
C6	0.0501 (9)	0.0949 (14)	0.0561 (10)	−0.0121 (9)	0.0413 (9)	−0.0238 (9)
C7	0.0496 (9)	0.0517 (10)	0.0764 (12)	−0.0062 (8)	0.0407 (9)	−0.0144 (9)
C8	0.0389 (8)	0.0609 (10)	0.0589 (9)	0.0083 (7)	0.0346 (8)	0.0143 (8)
C31	0.0324 (6)	0.0485 (8)	0.0387 (7)	−0.0004 (6)	0.0267 (6)	−0.0020 (6)
C32	0.0381 (7)	0.0542 (9)	0.0436 (8)	0.0022 (6)	0.0281 (7)	0.0033 (6)
C33	0.0401 (8)	0.0783 (12)	0.0377 (8)	−0.0057 (8)	0.0242 (7)	−0.0019 (7)
C34	0.0540 (9)	0.0730 (12)	0.0513 (9)	−0.0205 (9)	0.0375 (8)	−0.0225 (8)
C35	0.0561 (9)	0.0486 (9)	0.0644 (10)	−0.0069 (7)	0.0448 (9)	−0.0094 (7)
C36	0.0372 (7)	0.0492 (8)	0.0463 (8)	0.0002 (6)	0.0314 (7)	0.0001 (6)
C41	0.0424 (7)	0.0478 (8)	0.0458 (8)	0.0049 (6)	0.0319 (7)	0.0033 (6)
C42	0.0642 (10)	0.0543 (10)	0.0572 (10)	0.0153 (8)	0.0450 (9)	0.0058 (8)
C43	0.0912 (14)	0.0414 (9)	0.0548 (10)	0.0062 (9)	0.0469 (11)	0.0019 (7)
C44	0.0717 (12)	0.0561 (10)	0.0462 (9)	−0.0214 (9)	0.0364 (9)	−0.0070 (8)
C45	0.0528 (10)	0.0733 (12)	0.0604 (10)	−0.0111 (9)	0.0406 (9)	−0.0002 (9)
C46	0.0474 (9)	0.0530 (9)	0.0646 (10)	0.0060 (7)	0.0412 (8)	0.0072 (8)

### Geometric parameters (Å, °)

**Table:**

O1—C1	1.4437 (16)	O2—C5	1.4464 (17)
O1—H1	0.8400	O2—H2	0.8399
C1—C2	1.527 (2)	C5—C7	1.523 (2)
C1—C3	1.531 (2)	C5—C6	1.529 (2)
C1—C11	1.5364 (19)	C5—C31	1.540 (2)
C2—H2A	0.9800	C6—H6A	0.9800
C2—H2B	0.9800	C6—H6B	0.9800
C2—H2C	0.9800	C6—H6C	0.9800
C3—H3A	0.9800	C7—H7A	0.9800
C3—H3B	0.9800	C7—H7B	0.9800
C3—H3C	0.9800	C7—H7C	0.9800
C4—C21	1.512 (2)	C8—C41	1.515 (2)
C4—C16	1.522 (2)	C8—C36	1.524 (2)
C4—H4A	0.9900	C8—H8A	0.9900
C4—H4B	0.9900	C8—H8B	0.9900
C11—C12	1.3881 (19)	C31—C32	1.397 (2)
C11—C16	1.4018 (19)	C31—C36	1.403 (2)
C12—C13	1.386 (2)	C32—C33	1.387 (2)
C12—H12	0.9500	C32—H32	0.9500
C13—C14	1.364 (2)	C33—C34	1.375 (3)
C13—H13	0.9500	C33—H33	0.9500
C14—C15	1.378 (2)	C34—C35	1.377 (3)
C14—H14	0.9500	C34—H34	0.9500
C15—C16	1.396 (2)	C35—C36	1.394 (2)
C15—H15	0.9500	C35—H35	0.9500
C21—C26	1.363 (2)	C41—C42	1.384 (2)
C21—C22	1.374 (2)	C41—C46	1.389 (2)
C22—C23	1.397 (3)	C42—C43	1.390 (3)
C22—H22	0.9500	C42—H42	0.9500
C23—C24	1.372 (3)	C43—C44	1.377 (3)
C23—H23	0.9500	C43—H43	0.9500
C24—C25	1.357 (4)	C44—C45	1.368 (3)
C24—H24	0.9500	C44—H44	0.9500
C25—C26	1.350 (3)	C45—C46	1.382 (2)
C25—H25	0.9500	C45—H45	0.9500
C26—H26	0.9500	C46—H46	0.9500
			
C1—O1—H1	109.4	C5—O2—H2	109.3
O1—C1—C2	106.57 (12)	O2—C5—C7	107.22 (12)
O1—C1—C3	106.50 (13)	O2—C5—C6	106.36 (13)
C2—C1—C3	108.58 (14)	C7—C5—C6	108.63 (14)
O1—C1—C11	110.01 (11)	O2—C5—C31	109.39 (11)
C2—C1—C11	111.55 (12)	C7—C5—C31	113.51 (13)
C3—C1—C11	113.30 (12)	C6—C5—C31	111.40 (12)
C1—C2—H2A	109.5	C5—C6—H6A	109.5
C1—C2—H2B	109.5	C5—C6—H6B	109.5
H2A—C2—H2B	109.5	H6A—C6—H6B	109.5
C1—C2—H2C	109.5	C5—C6—H6C	109.5
H2A—C2—H2C	109.5	H6A—C6—H6C	109.5
H2B—C2—H2C	109.5	H6B—C6—H6C	109.5
C1—C3—H3A	109.5	C5—C7—H7A	109.5
C1—C3—H3B	109.5	C5—C7—H7B	109.5
H3A—C3—H3B	109.5	H7A—C7—H7B	109.5
C1—C3—H3C	109.5	C5—C7—H7C	109.5
H3A—C3—H3C	109.5	H7A—C7—H7C	109.5
H3B—C3—H3C	109.5	H7B—C7—H7C	109.5
C21—C4—C16	115.49 (14)	C41—C8—C36	113.72 (13)
C21—C4—H4A	108.4	C41—C8—H8A	108.8
C16—C4—H4A	108.4	C36—C8—H8A	108.8
C21—C4—H4B	108.4	C41—C8—H8B	108.8
C16—C4—H4B	108.4	C36—C8—H8B	108.8
H4A—C4—H4B	107.5	H8A—C8—H8B	107.7
C12—C11—C16	117.75 (13)	C32—C31—C36	118.29 (13)
C12—C11—C1	119.36 (12)	C32—C31—C5	119.27 (14)
C16—C11—C1	122.89 (12)	C36—C31—C5	122.44 (13)
C13—C12—C11	122.68 (14)	C33—C32—C31	121.87 (16)
C13—C12—H12	118.7	C33—C32—H32	119.1
C11—C12—H12	118.7	C31—C32—H32	119.1
C14—C13—C12	119.51 (14)	C34—C33—C32	119.43 (16)
C14—C13—H13	120.2	C34—C33—H33	120.3
C12—C13—H13	120.2	C32—C33—H33	120.3
C13—C14—C15	118.97 (14)	C33—C34—C35	119.54 (16)
C13—C14—H14	120.5	C33—C34—H34	120.2
C15—C14—H14	120.5	C35—C34—H34	120.2
C14—C15—C16	122.58 (15)	C34—C35—C36	122.06 (17)
C14—C15—H15	118.7	C34—C35—H35	119.0
C16—C15—H15	118.7	C36—C35—H35	119.0
C15—C16—C11	118.50 (13)	C35—C36—C31	118.74 (14)
C15—C16—C4	118.95 (14)	C35—C36—C8	116.63 (15)
C11—C16—C4	122.44 (13)	C31—C36—C8	124.63 (14)
C26—C21—C22	118.66 (16)	C42—C41—C46	117.88 (15)
C26—C21—C4	121.96 (18)	C42—C41—C8	120.72 (14)
C22—C21—C4	119.35 (17)	C46—C41—C8	121.37 (14)
C21—C22—C23	120.08 (17)	C41—C42—C43	120.72 (17)
C21—C22—H22	120.0	C41—C42—H42	119.6
C23—C22—H22	120.0	C43—C42—H42	119.6
C24—C23—C22	119.11 (19)	C44—C43—C42	120.39 (17)
C24—C23—H23	120.4	C44—C43—H43	119.8
C22—C23—H23	120.4	C42—C43—H43	119.8
C25—C24—C23	120.03 (18)	C45—C44—C43	119.46 (17)
C25—C24—H24	120.0	C45—C44—H44	120.3
C23—C24—H24	120.0	C43—C44—H44	120.3
C26—C25—C24	120.5 (2)	C44—C45—C46	120.31 (17)
C26—C25—H25	119.8	C44—C45—H45	119.8
C24—C25—H25	119.8	C46—C45—H45	119.8
C25—C26—C21	121.7 (2)	C45—C46—C41	121.23 (16)
C25—C26—H26	119.2	C45—C46—H46	119.4
C21—C26—H26	119.2	C41—C46—H46	119.4
			
O1—C1—C11—C12	129.43 (14)	O2—C5—C31—C32	131.82 (13)
C2—C1—C11—C12	−112.53 (16)	C7—C5—C31—C32	12.12 (18)
C3—C1—C11—C12	10.4 (2)	C6—C5—C31—C32	−110.88 (16)
O1—C1—C11—C16	−51.21 (18)	O2—C5—C31—C36	−47.57 (17)
C2—C1—C11—C16	66.83 (18)	C7—C5—C31—C36	−167.27 (13)
C3—C1—C11—C16	−170.28 (15)	C6—C5—C31—C36	69.72 (18)
C16—C11—C12—C13	0.9 (2)	C36—C31—C32—C33	1.1 (2)
C1—C11—C12—C13	−179.75 (15)	C5—C31—C32—C33	−178.27 (13)
C11—C12—C13—C14	0.3 (3)	C31—C32—C33—C34	1.2 (2)
C12—C13—C14—C15	−1.2 (3)	C32—C33—C34—C35	−2.2 (2)
C13—C14—C15—C16	1.1 (3)	C33—C34—C35—C36	0.9 (2)
C14—C15—C16—C11	0.1 (3)	C34—C35—C36—C31	1.4 (2)
C14—C15—C16—C4	−176.00 (18)	C34—C35—C36—C8	−179.60 (14)
C12—C11—C16—C15	−1.0 (2)	C32—C31—C36—C35	−2.39 (19)
C1—C11—C16—C15	179.59 (14)	C5—C31—C36—C35	177.01 (12)
C12—C11—C16—C4	174.93 (18)	C32—C31—C36—C8	178.73 (12)
C1—C11—C16—C4	−4.4 (3)	C5—C31—C36—C8	−1.9 (2)
C21—C4—C16—C15	−17.7 (3)	C41—C8—C36—C35	51.16 (19)
C21—C4—C16—C11	166.40 (17)	C41—C8—C36—C31	−129.93 (15)
C16—C4—C21—C26	−81.9 (2)	C36—C8—C41—C42	−129.26 (16)
C16—C4—C21—C22	100.1 (2)	C36—C8—C41—C46	52.9 (2)
C26—C21—C22—C23	−0.2 (2)	C46—C41—C42—C43	0.1 (3)
C4—C21—C22—C23	177.91 (14)	C8—C41—C42—C43	−177.79 (16)
C21—C22—C23—C24	0.8 (2)	C41—C42—C43—C44	0.2 (3)
C22—C23—C24—C25	−0.3 (3)	C42—C43—C44—C45	−0.5 (3)
C23—C24—C25—C26	−0.7 (3)	C43—C44—C45—C46	0.3 (3)
C24—C25—C26—C21	1.3 (3)	C44—C45—C46—C41	0.1 (3)
C22—C21—C26—C25	−0.8 (2)	C42—C41—C46—C45	−0.3 (3)
C4—C21—C26—C25	−178.88 (16)	C8—C41—C46—C45	177.62 (16)

### Hydrogen-bond geometry (Å, °)

**Table:**

*D*—H···*A*	*D*—H	H···*A*	*D*···*A*	*D*—H···*A*
O1—H1···O2^i^	0.84	1.98	2.7997 (14)	166.
O2—H2···O1	0.84	1.94	2.7486 (14)	161.

Symmetry codes: (i) −*x*+1, −*y*, −*z*.
